# Reverse re-modelling chronic heart failure by reinstating heart rate variability

**DOI:** 10.1007/s00395-022-00911-0

**Published:** 2022-02-01

**Authors:** J. Shanks, Y. Abukar, N. A. Lever, M. Pachen, I. J. LeGrice, D. J. Crossman, A. Nogaret, J. F. R. Paton, R. Ramchandra

**Affiliations:** 1 Manaaki Manawa—The Centre for Heart Research, Department of Physiology, University of Auckland, Park Road, Grafton, Auckland, New Zealand; 2 Department of Cardiology, Auckland City Hospital, Auckland District Health Board, Park Road, Grafton, Auckland, New Zealand; 3 Department of Physics, University of Bath, Claverton Down, Bath, UK

**Keywords:** Heart failure, Respiratory sinus arrhythmia, Cardiac output, Pacemaker

## Abstract

Heart rate variability (HRV) is a crucial indicator of cardiovascular health. Low HRV is correlated with disease severity and mortality in heart failure. Heart rate increases and decreases with each breath in normal physiology termed respiratory sinus arrhythmia (RSA). RSA is highly evolutionarily conserved, most prominent in the young and athletic and is lost in cardiovascular disease. Despite this, current pacemakers either pace the heart in a metronomic fashion or sense activity in the sinus node. If RSA has been lost in cardiovascular disease current pacemakers cannot restore it. We hypothesized that restoration of RSA in heart failure would improve cardiac function. Restoration of RSA in heart failure was assessed in an ovine model of heart failure with reduced ejection fraction. Conscious 24 h recordings were made from three groups, RSA paced (*n* = 6), monotonically paced (*n* = 6) and heart failure time control (*n* = 5). Real-time blood pressure, cardiac output, heart rate and diaphragmatic EMG were recorded in all animals. Respiratory modulated pacing was generated by a proprietary device (Ceryx Medical) to pace the heart with real-time respiratory modulation. RSA pacing substantially increased cardiac output by 1.4 L/min (20%) compared to contemporary (monotonic) pacing. This increase in cardiac output led to a significant decrease in apnoeas associated with heart failure, reversed cardiomyocyte hypertrophy, and restored the T-tubule structure that is essential for force generation. Re-instating RSA in heart failure improves cardiac function through mechanisms of reverse re-modelling; the improvement observed is far greater than that seen with current contemporary therapies. These findings support the concept of re-instating RSA as a regime for patients who require a pacemaker.

## Introduction

The heart beats with a number of physiological rhythms including synchronization between the heart chambers, variations in heartbeat intervals, and altered resting heart rate across the day [15]. Heart rate variability (HRV) refers to the period between heartbeats, and in a healthy individual, this is not constant [53]. In 1847, Ludwig first realized that breathing modulated the heartbeat frequency, which was termed respiratory sinus arrhythmia (RSA) [9]. RSA is a highly evolutionary conserved phenomenon, most prominent in the young and indicative of physical fitness [14, 18, 22]. In cardiovascular disease, RSA declines [18, 53] and is lost in patients with heart failure [18]. The loss of RSA is a prognostic indicator for many diseases including sudden cardiac death [27]. While the presence of RSA for health is undisputed [14, 19, 22, 53] the function(s) of RSA remain debated [7, 19, 20, 39, 54, 55].

Heart failure affects around 26 million people worldwide and around 50% of patients die within 5 years of diagnosis [32]. While currently available pharmacological treatments are effective in most patients, device therapy is considered in patients who remain symptomatic despite optimal use of medicines [43]. The last century has seen great advances in pacemaker design, from device miniaturization, to bi-ventricular pacing with cardiac resynchronization therapy [1, 52], and the introduction of underlying facilitating currents with cardiac contractility modulation [2, 28]. However, all pacemakers to date generate an output with no breath-by-breath induced variation in the inter-beat interval. Given the conserved evolutionary importance of RSA, and its proposed cardiac energy-saving function [8], we tested the hypothesis that re-instating RSA would improve cardiac function in a clinically translatable ovine model of heart failure with reduced ejection fraction.

## Materials and methods

### Animals

**Fig. 1:**
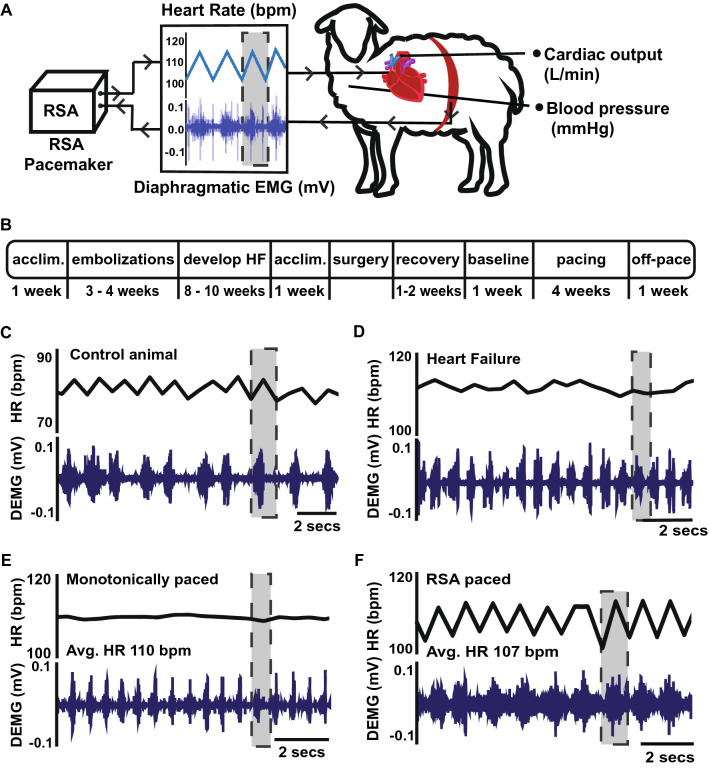
**A**. Schematic representation of the experimental set-up. Fully instrumented conscious 3–5 year old, outbred, female Romney sheep were paced via the left atrium, 1.5–2.5 V, using a neuron-based pace making device that modulates heart rate in phase with respiration on a breath by breath basis mimicking respiratory sinus arrhythmia (RSA). The device receives diaphragmatic EMG (electromyogram) input to define the phase of inspiration. RSA increases heart rate during inspiration as indicated by the highlighted regions in panels A, **C**–**F** Cardiac output was measured on a beat by beat basis using an implantable flow probe attached to the ascending aorta. **B** Time-line of the six-month experimental protocol. Heart failure with reduced ejection fraction was induced by microembolization and followed by an 8–10 week holding period for chronic heart failure to develop prior to pacing. Following instrumentation and stabilization of hemodynamics parameters, animals were divided into three groups: two were paced (RSA or monotonically) and one not paced (time control) for 4 weeks. This was followed by 1 week off pacing before animals were anaesthetized for baroreflex testing and cardiac tissue collected for post hoc analysis. **C** Representative raw data traces from a healthy sheep showing native RSA. **D** A heart failure sheep showing loss of RSA. **E** A heart failure sheep paced monotonically, showing a stable heart rate. **F** A heart failure sheep being RSA paced. Note that there was no difference between the mean heart rate of monotonically and RSA paced sheep. *Acclim* acclimatization to the laboratory; *Avg.* average; bpm, beats per minute; *DEMG* diaphragmatic electromyogram; *HR* heart rate; *mV* milli Volts

Adult (3–5 year old) female Romney sheep were housed in individual crates, and acclimatized to laboratory conditions (18 °C, 50% relative humidity, 12 h light–dark cycle) and human contact for 1 week before any experiments. Sheep were fed 2–2.5 kg/day (Country harvest pellets) and had access to water ad libitum. All animal experiments and surgical procedures followed relevant guidelines and were approved by the Animal Ethics Committee of the University of Auckland (#2082). Experiments were carried out over a period of 6 months in each animal; a summary is schematized in Fig. 1B.

### Induction of heart failure—Embolization Surgical Procedure

Three groups of heart failure sheep were studied: RSA, monotonically paced, and a time control. In the conscious state and standing, echocardiography was performed (Phillips HD-11 Ultrasound) to measure ejection fraction in the healthy animals (typically 70–80%). Subsequently, sheep were anaesthetized which was induced with 2% Diprivan (Propofol) (5 mg/kg i.v. AstraZeneca, AUS), maintained with a 2% isoflurane-air-O_2_ mixture and were intubated for mechanical ventilation. Anaesthesia depth was monitored throughout the surgery by an absence of the corneal reflex and an absence of a withdrawal response to a noxious pinch. The left or right femoral artery was accessed percutaneously using an 8F (CORDIS®, USA) sheath and under fluoroscopic guidance, the left main coronary artery was then cannulated, and the catheter advanced into either the proximal left main coronary artery or left descending coronary arteries as described before [4]. To induce heart failure with reduced ejection fraction sheep underwent sequential weekly (1–3 weeks) embolizations using polystyrene latex microspheres (45 μm; 1.2 mL, approximately 650 microspheres, Polysciences, Warrington, PA, USA). Prior to the injection of microspheres, β-blocker (metoprolol up to 20 mg/kg, IV) and lignocaine (2 mg/kg, IV) were injected intravenously to prevent ventricular arrhythmias. Electrocardiogram (ECG) was recorded from lead II prior to the infusion of the microspheres and for a further 5 min after infusion. The recordings were made on a dual bio amp electrocardiograph switch box with a power lab and LabChart (AD Instruments, NZ). A change in the ST segment (elevation or depression) and T wave (inversion) was taken as an indication of a successful embolization.

Three days post embolization, conscious sheep underwent echocardiography; once ejection fraction dropped from 70 to 80% to ~ 45%, no more embolizations were performed. A drop in ejection fraction to ~ 45% was selected as the criteria for successful induction of heart failure. Giving all sheep the maximum three embolizations without taking into account ejection fraction resulted in unnecessary animal loss in previous studies. There was a loss of three animals during or within 48 h post the embolizations in this study. Echocardiograms were repeated 1 week, and 3 months post embolization, then once weekly during pacing and the post-pace period. In the long-axis M-mode, diastole, systole, fractional shortening, and ejection fraction were obtained and calculated for the left ventricle. There were no differences in body weight or baseline ejection fraction between groups at the start of the experiment.

### Instrumentation surgery

Three months after heart failure was induced by microembolization, left ventricle ejection fraction was again checked using echocardiography. No sheep was found to significantly increase in ejection fraction over the 3 months and there was a loss of one animal during this period. Once confirmed to be ~ 45%, sheep underwent instrumentation under general anaesthesia (induced with 2% Diprivan (Propofol) 5 mg/kg i.v., AstraZeneca, AUS) and maintained with a 2% isoflurane-air-O_2_ mixture. An intercostal nerve block was performed on ribs 2–6, using a 22G needle 1 mL sterile saline followed by 3 mL Bupivavaine which was injected into the intercostal space immediately cranially of each rib. Sheep were placed on their right side and instrumentation was carried out in full sterile conditions.

A 10 cm incision was made on the left side of the neck. To get an index of blood pressure a single-tip pressure probe (Millar Inc., Texas, USA) was inserted into the left common carotid artery. Cannulae were inserted into both the jugular vein for venous infusion and the common carotid artery for arterial blood sampling. The pressure probe and catheters were secured with a purse-string suture (Filasilk, 3.0 non-absorbable braided silk suture) to maintain blood flow through the vessel. The incision in the neck was closed with 1.0 suture.

To gain access to the heart a dorsal to ventral incision was made on the left side of the chest with a scalpel (size 20) and diathermy (Aaron 250, Bovie Medical), muscle layers were separated and either the fourth or fifth rib was removed. The chest was opened with a retractor and the pericardial sack opened. To measure cardiac output the aorta was separated from the pulmonary artery, and a doppler flow probe (Size 22, Transonic, AU) was placed around the ascending aorta for a direct measure of cardiac output on a beat-by-beat basis. For cardiac pacing two pacing leads (Biotonik, Berlin, Germany, Solia S 53- in case of failure in one) were secured externally to the left atrium with 3.0 Filasilk suture and silicone gel. The pericardial sack was closed with 3.0 Filasilk suture. To gain a real time measure of respiration electrodes were implanted into the diaphragm to give a measure of diaphragmatic EMG (‘DEMG’) as previously described [21]. Two strips of seven-stranded Cooner Wires (AS 633-7SSF, Cooner Wire, CA, USA) were passed through the diaphragm using a needle and an exposed section of the wire was in contact with the diaphragm and secured with silicone gel.

The chest was closed using sutures in each of the tissue layers including the intercostal muscles (Covidien, Surgipro, 1.0 monofilament polypropylene suture) and skin (1.0 braided silk suture). Negative pressure was re-established in the chest, flow probe, pacing and DEMG leads were tunnelled sub-cutaneously and exited percutaneously on the dorsum of the sheep for connecting to chronic recording devices after recovery.

Sheep were given antibiotic injections (6 mL i.m.; Oxytetra, Phenix, NZ), and analgesia (Ketofen 10%, 1 mL i.m.; Merial, Boehringer Ingelheim, NZ) at the start of surgery, and for the first 3 days post-operatively. Animals were allowed to recover for 5–7 days.

### Hemodynamic measurements and analysis

All analyses were repeated independently by two experienced members of the group. Cardiovascular and respiratory parameters were recorded from conscious sheep in heart failure on a desktop computer with a CED micro 1401 interface and a data acquisition program (Spike2 v8, Cambridge Electronic Design, UK). A baseline recording period was acquired when heart rate and cardiac output had stabilized post-operatively (between 5 and 7 days). Continuous arterial blood pressure, cardiac output from the ascending aorta blood flow probe and heart rate were recorded 24 h per day for 5 weeks. Heart rate was calculated from the inter-pulse interval of the blood flow in the ascending aorta. DEMG signal was amplified (X10, 000), and filtered (band pass 0.3–3.0 kHz).

### Pacing protocols

Sheep were randomized into three different groups before instrumentation surgery. There was a loss of four animals in total (out of 24) during the instrumentation surgery but this was not different between the groups. The first three animals were time controls, following which animals were randomly assigned to the different groups. The sinusoidal group was conducted once the results from the RSA and monotonic groups were collected.

#### Monotonic—rate fixed pacing (*n* = 6)

Pacing leads were connected to a stimulator (Grass Instruments) and pacing was set at 10–15 beats per minute above the resting heart rate of each sheep (Fig. 1E). To achieve the desired heart rate the frequency of the stimulation pulse (1.5–2.5 V, 2 ms pulse width) was adjusted and visualized in Spike2 before connecting to the pacing lead of the animal. Pacing voltage was adjusted during the 4 weeks pacing period if pacing became intermittent.

#### RSA pacing (*n* = 6)

RSA pacing was achieved using a biofeedback device described previously [37–39]. This device performed real-time integration of the diaphragmatic electromyographic (EMG) activity [38], used to define the inspiratory phase (Ceryx Medical, UK; Fig. 1A). The device exploits the excitatory response of neuronal oscillators to increase and decrease heart rate during inspiration and expiration, respectively (Fig. 1A, F) [36, 37]. DEMG input was split to enable both raw DEMG to be recorded and provide an input into the pacing device. Pacing was set 10–15 beats per minute above resting heart rate with an RSA magnitude (peak-to-trough) of 12 beats per minute. Pacing voltage was set at 1.5 V and pulse width at 2 ms. Pacing voltage was increased if and as needed during the four-week pacing period. RSA pacing was visually checked against the DEMG channel to ensure the rising phase of heart rate correlated with inspiration, and the falling phase of heart rate with expiration (Fig. 1F).

#### Time control—no pacing (*n* = 5)

**Fig. 2:**
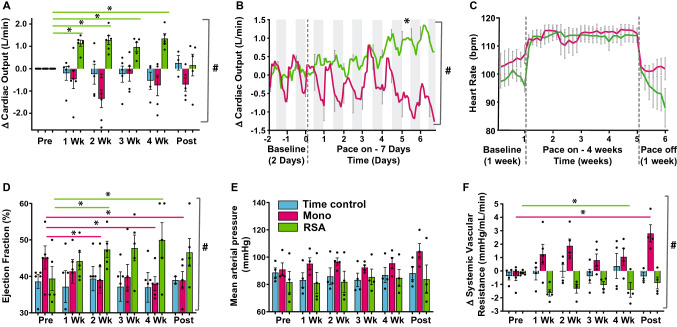
Data were collected for 24 h a day for 6 weeks (1 week baseline or pre-pacing, 4 weeks pacing; 1 week pace off post-pacing) from chronically instrumented, conscious sheep. **A** Cardiac output was measured directly on a beat by beat basis and each data point represents the 24 h average change in cardiac output. Monotonically paced (Mono: magenta, *n* = 6 paced to day 14, *n* = 5 paced for 4 weeks) and respiratory sinus arrhythmia paced (RSA: green, *n* = 6 paced to day 14, *n* = 5 paced for 4 weeks) were paced for 28 days. A time control group (TC: blue, *n* = 5 to day 32, *n* = 3–4 weeks) was not paced. **B** Cardiac output change from baseline on expanded time scale over first week of pacing. Each data point represents 2 h average cardiac output. Mono + RSA (*n* = 6 each) animals only. **C** Mean heart rate over time; each data point is a 24 h average (Mono: *n* = 6 paced to day 14, *n* = 5 paced for 4 weeks. RSA: *n* = 6 paced to day 14, *n* = 5 paced for 4 weeks. TC: *n* = 5 to day 32, *n* = 3–4 weeks). **D** Ejection fraction measured using echocardiography from conscious sheep (TC, *n* = 2–5, Mono *n* = 5, RSA *n* = 5). **E** Mean arterial pressure (all groups, *n* = 4). **F** Systemic vascular resistance (all groups, n = 4). Note that the Posthoc test significance for the RSA group week 4 vs. baseline was *P* = 0.057 For **D–F,** all data are presented as a 24 h average. 2-way ANOVA interaction effect; #*P* < 0.05. Posthoc analysis (Dunnetts) to compare the time points to baseline;*P < 0.05

Time control animals were set up identically to the other two groups but had no pacemaker connected to the pacing lead. Our protocols ensured that both the RSA and Mono paced animals received the same number of heartbeats (Fig. 2C).

#### Pacing under periodic modulation (*n* = 3)

Variable heart rate pacing, not phase locked to respiration, was achieved by feeding a steady-state square wave signal as the input into the RSA pacing device (instead of the animal DEMG input). Frequency was set to produce a peak-to-tough amplitude of 12 bpm, comparable to the RSA group (stimulation pulse 1.5–2.0 V, 2 ms pulse width).

### Pacing efficacy

To determine the percentage of the day the animals were being paced, pacing efficacy was calculated. For monotonically paced sheep the number of heartbeats with a beat interval outside the target paced range was calculated as a percentage of the total number of heart beats. For RSA paced animals, a threshold was placed on the heart rate channel at a value just below the pre-set peak heart rate change during inspiration. This value was subtracted from the total number of breaths to give RSA pacing efficacy.

### Breath rate and apnoea analysis

Breathing parameters were assessed from 24 h DEMG recordings. Breath rate was determined as average breaths per minute over 24 h. Apnoea was defined as cessation of diaphragmatic activity. Apnoea incidence was determined by calculating the total number of apnoeas of given lengths (> 3 secs, > 4 secs, > 5 secs, > 6 secs) in 24 h.

### Plasma brain natriuretic peptide measurement

Arterial blood samples (20 mL) were collected into EDTA tubes (BD Vacutainer, NJ, USA). Plasma was separated from whole blood by centrifugation (4 °C, 3000 rpm, 15 min), rapidly frozen and stored at − 80 °C before processing. All samples were coded and measurements were made in blinded fashion by the Christchurch Heart Institute; results were returned for decoding. The assay for brain natriuretic peptide has been described previously.

### Direct recordings of renal sympathetic nerve activity

An incision was made in the left flank and the left renal artery and renal nerve exposed using methods routine in the lab. Renal sympathetic nerve activity (RSNA) was differentially recorded between a pair of electrodes. The signal was amplified (× 20,000) and filtered (bandpass 400–1200 Hz). Mean arterial pressure was obtained by the pressure probe already implanted. Changes in blood pressure and renal sympathetic nerve active were measured in response to phenylephrine and sodium nitroprusside. For both drugs, the concentration increased at 1-min intervals and doses were 25, 50, 100, 200, and 400 mg/min. All the parameters were recorded on a desktop computer with a CED micro 1401 interface and a data acquisition program (Spike2 v8, Cambridge Electronic Design, UK).

At the end of these experiments, the sheep were euthanized with an overdose of sodium pentobarbitone (0.5 mL/kg, intravenously) (Provet NZ Pty Ltd., New Zealand). Once death was confirmed, heart and lungs were weighed, in relation to tibia length and cardiac tissue collected for further analysis.

### Tissue histology

All tissue samples were collected from the LV mid-free wall, approximately half-way between the atria and the apex. Care was taken to ensure all tissue samples collected were from the same location between animals, and, therefore, should represent a similarly ischemic area of tissue.

### Cell membrane staining

Freshly dissected samples from the LV were cut into 0.5 cm thick 1 cm strips spanning the epi-to endocardial surfaces. Samples were collected from all sheep from the same region of the LV. Prior to fixation samples were placed in Tyrode’s solution containing BDM to prevent contraction artifacts from rapid cooling. LV samples were fixed in 1% paraformaldehyde for 1 h 4 °C, before being moved through three solutions of increasing sucrose gradient (10%, 20%, 30%) over 48 h. Once cryoprotected with sucrose, tissue samples were frozen in liquid nitrogen chilled methylbutane (Thermofisher), and stored at -80 °C before processing.

Fixed frozen samples were cut into 12 µm sections on a Leica CM 1900 cryostat and mounted on Superfrost Plus cover slips. Slides were rinsed with phosphate-buffered saline (PBS), and incubated in Fx signal enhancer (Thermofisher) for 1 h at room temperature. Slides were then washed 3 × with PBS, followed by incubating in a 1:100 concentration of wheat-germ agglutinin (WGA, Alexa Fluor 594, Fisher. cat# W11262) for 2 h at room temp. Slides were again washed 3 × with PBS before applying VectaShield and cover slipping. All analysis of cell size and t-tubule power was conducted by experienced members blinded to the group status.

#### Cell size

Fluorescent images of labelled tissue sections were imaged on an Olympus FV1000 confocal microscope (diode-pumped 559 nm laser, 20×/0.8 NA oil, FluoView 4.2). 5–8 different regions from each stained LV slice were imaged at 20×. Analysis was performed using ImageJ (Fiji). A two-pixel median filter was applied to all images. Individual cardiomyocytes deemed to be lying parallel to the imaging, in full longitudinal view were traced around the circumference to measure cell area (µm^2^). For each region, 5–10 different cells were measured and an average cell size presented.

#### T-tubule power

T-tubule power was determined by previously described methods. The WGA stained LV samples were imaged on an Olympus FV1000 confocal microscope (diode-pumped 559 nm laser, 60×/1.35 NA oil, FluoView 4.2) to a pixel size of 80 nm. 10–12 areas were imaged at 60 × per LV section. Images were analyzed in ImageJ (Fiji). All images underwent a 2 pixel median filter, and 0.3 pixel background subtract. FFT was performed along a selected 20 µm line (Extended Data 3). 2–4 individual myocytes were analyzed per image. T-tubule power was determined from a power spectrum obtained from the FFT.

### Statistical analysis

All data are expressed as mean ± SEM, except where indicated. All-time course data (cardiac output, heart rate, mean arterial pressure, systemic vascular resistance, stroke volume, breath rate) were analyzed using a repeated measures 2-way ANOVA, or mixed-effects model if any missed data points were ‘missing at random’. Posthoc analysis was completed if there was an effect of time and Dunnetts two-sided post hoc tests were used to compare the time points to baseline. Renal sympathetic nerve recordings were analyzed in SigmaPlot. Heart weight, and lung weight were analyzed between monotonic and RSA paced sheep using an unpaired two-tailed students *t*-test. Histology analysis was analyzed between three groups, time control, monotonic paced, and RSA paced using an ordinary one-way ANOVA. All statistical analysis was performed in SPSS (v8.1)**.** Data were considered significant if *p* < 0.05.

## Results

### Re-instating RSA in heart failure

In fully instrumented conscious sheep with chronic heart failure, we used a novel neuron-based pace-making device [36] to modulate heart rate on a breath-by-breath basis mimicking RSA for 4 weeks (Fig. 1A, B). We compared changes in cardiac output between three groups of sheep with heart failure (5–6 sheep per group); sheep receiving RSA (Fig. 1F) versus conventional metronomic pacing (Fig. 1E) and no pacing (Fig. 1D), which served as a time control. Reinstatement of RSA substantially increased cardiac output by 1.4 ± 0.5 L/min compared to both Mono and non-paced time control (TC) sheep with heart failure (*P* < 0.001, interaction factor; Fig. 2A, B). The increase in cardiac output reached its peak effect after 5 ± 1.3 days of pacing and was sustained over the 4 weeks of pacing (Fig. 2A). The increase in cardiac output was not immediate (Fig. 2A, B) suggesting that this was not a direct haemodynamic effect. Similarly, switching off the pacemaker did not result in an abrupt reduction in cardiac output back to baseline; rather this reduced slowly over a week to its pre-RSA pacing level (Fig. 2A). This result indicates that the effect of RSA pacing can persist for several days without RSA being present. There were no significant differences in baseline parameters between the Mono, RSA or the time control groups (Table 1). In particular, left ventricular diastolic volumes were not different between the groups at the start of the pacing protocol (148 ± 24 ml, Mono; 143 ± 30 ml, RSA; 151 ± 20 ml, TC). Echocardiography revealed that RSA increased ejection fraction (*P* < *0.05*; Fig. 2D), stroke volume (*P* < *0.05*) and fractional shortening (*P* < *0.01*; Table 1) from baseline; Mono pacing reduced ejection fraction (*P* < *0.05*; Fig. 2D), stroke volume (*P* < *0.05*) and fractional shortening (*P* < *0.01*; Table 1) from baseline, and there was no changes in these parameters in the heart failure TC groups.

**Table 1:** Raw data values of ejection fraction, stroke volume and fractional shortening obtained using echocardiography on the three groups (time control, monotonically and respiratory sinus arrhythmia (RSA) paced) of conscious heart failure sheep

Stroke volume (mL)			
	Heart Failure Time-control (*n* = 5)	Heart Failure Monotonic-pacing (*n* = 5)	Heart Failure RSA-pacing (*n* = 5)
Baseline	60.9 ± 23.9	78.7 ± 12.3	78.1 ± 14.5
2 weeks	63.2 ± 28.1	58.2 ± 12.7*	73.9 ± 16.1
4 weeks	64.8 ± 29.1	53.8 ± 12.2*	79.0 ± 16.0
Post-pace	65.9 ± 31.8	62.7 ± 14.8*	88.9 ± 15.8*

Fractional shortening (%)			
	Heart Failure Time-control (*n* = 5)	Heart Failure Monotonic-pacing (*n* = 5)	Heart Failure RSA-pacing (*n* = 5)
Baseline	18.0 ± 2.6	23.9 ± 5.8	18.7 ± 5.0
2 weeks	17.2 ± 1.3	20.6 ± 5.9	26.8 ± 5.1*
4 weeks	16.3 ± 2.9*	18.7 ± 2.6*	28.0 ± 8.0*
Post-pace	15.9 ± 2.6*	18.7 ± 2.6*	25.8 ± 6.7 *

	Heart Failure Monotonic-pacing (*n* = 3)	Heart Failure RSA-pacing (*n* = 3)
Heart weight (g)	486 ± 21.7	506 ± 12.7
Lung weight (g)	634 ± 45.0	612 ± 31.8
Tibia length (cm)	18.4 ± 0.23	18.3 ± 0.15
Heart weight/tibia length (cm/g)	26.5 ± 0.93	27.6 ± 0.85
Lung weight/tibia length (cm/g)	34.5 ± 2.01	33.4 ± 1.75

Heart and lung wet weights are indicated and corrected against tibia length [57]

*P < 0.05 Post-hoc Dunnetts test baseline to each time point

Next, we quantified the amount of RSA pacing delivered to the sheep. We calculated that RSA pacing was present for 42 ± 14% of each day. When not being paced with RSA, the pacemaker automatically paces monotonically at a rate equivalent to the mean rate between the peak/trough values of RSA pacing. For the Mono group, pacing was achieved 95% of the time per day.

### Blood pressure and its baroreflex control and autonomic balance

In sheep with RSA pacing, arterial pressure was unchanged (Fig. 2E and Table 2). The elevated CO with no change in arterial pressure means that calculated systemic vascular resistance decreased by 13 ± 6% (Fig. 2F; *P* < 0.01). In contrast, in Mono paced sheep, there was no change in arterial pressure but there was a tendency for CO to decrease which meant that calculated systemic vascular resistance actually increased (Fig. 2F; *P* < 0.05).

**Table 2:** Raw data values of cardiac output, mean arterial pressure, systemic vascular resistance, and heart rate from the three groups of conscious heart failure sheep (time control, monotonic and respiratory sinus arrhythmia (RSA) paced)

Cardiac output (L/min)			
	Heart Failure Time-control (*n* = 5)	Heart Failure Monotonic-pacing (*n* = 5)	Heart Failure RSA-pacing (*n* = 5)
Baseline	7.8 ± 1.3	8.7 ± 1.6	7.6 ± 1.3
2 weeks	7.7 ± 1.4	7.2 ± 2.1*	8.4 ± 1.5
4 weeks	7.7 ± 1.9	7.5 ± 2.0	8.9 ± 1.6*
Post-pace	7.3 ± 1.4	7.5 ± 1.9	7.7 ± 1.6

Mean arterial pressure (mmHg)			
	Heart Failure Time-control (*n* = 5)	Heart Failure Monotonic-pacing (*n* = 5)	Heart Failure RSA-pacing (*n* = 5)
Baseline	88.7 ± 6.7	91.1 ± 9.4	81.7 ± 15.6
2 weeks	86.0 ± 12.3	96.7 ± 5.5	81.9 ± 15.6
4 weeks	87.0 ± 11.1	95.6 ± 8.3	84.9 ± 12.2
Post-pace	88.3 ± 9.7	104.2 ± 11.4	83.9 ± 20.7

Systemic vascular resistance (mmHg/L)			
	Heart Failure Time-control (*n* = 4)	Heart Failure Monotonic-pacing (*n* = 4)	Heart Failure RSA-pacing (*n* = 4)
Baseline	11.6 ± 2.8	10.3 ± 1.9	10.3 ± 0.9
2 weeks	11.4 ± 2.7	13.2 ± 3.4	9.4 ± 1.5
4 weeks	12.0 ± 4.7	13.0 ± 2.2	9.2 ± 1.4
Post-pace	12.4 ± 5.3	14.0 ± 2.8*	10.2 ± 1.2

Heart rate (bpm)			
	Heart Failure Time-control (*n* = 5)	Heart Failure Monotonic-pacing (*n* = 5)	Heart Failure RSA-pacing (*n* = 5)
Baseline	110 ± 12	105 ± 7.0	98 ± 4.0
2 weeks	106 ± 7.5	114 ± 5.9	113 ± 6.3
4 weeks	105 ± 5.0	114 ± 5.6	114 ± 6.1
Post-pace	104 ± 4.2	102 ± 6.5	97 ± 10.3

*P < 0.05 Post-hoc Dunnetts test baseline to each time point

**Fig. 3:**
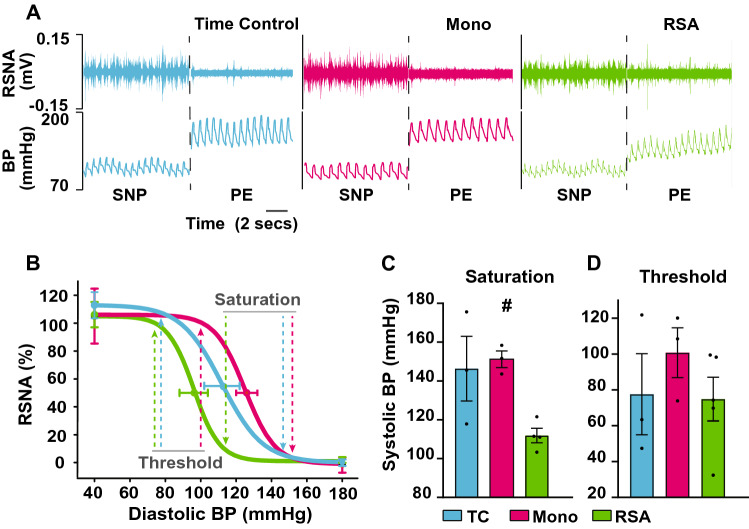
**A** Renal sympathetic nerve activity (RSNA) was recorded under general anesthesia at the end of the sixth week. Representative raw data traces of RSNA and blood pressure (BP) in response to a baroreflex challenge with phenylephrine (PE) and sodium nitroprusside (SNP). Time control (blue), Mono (magenta) and RSA paced (green). **B** RSNA baroreflex function curves for the three groups of sheep. **C** Baroreflex saturation is the systolic blood pressure when RSNA reaches zero percent of maximum. **D** Threshold is the point of the baroreflex curve when RSNA reaches a maximum at low-pressure levels (TC *n* = 3; Mono *n* = 3; RSA *n* = 4)

Under anaesthesia, baroreflex function was assessed at the end of the protocol (Fig. 1B) and arterial pressure was ramped up/down with vasoactive agents while recording renal sympathetic nerve activity in all three groups (Fig. 3A). Although there was no change in reflex sensitivity between groups, in RSA paced sheep the reflex function curve was left-shifted (Fig. 3A,B; *P* = 0.06) re-setting to operate over a lower blood pressure range, and baroreflex saturation (blood pressure level where sympathetic activity was fully inhibited) as significantly reduced (Fig. 3C; *P* < 0.05). In contrast, there was no difference in cardiac baroreflex gain between groups.

### Breathing instability in heart failure

**Fig. 4:**
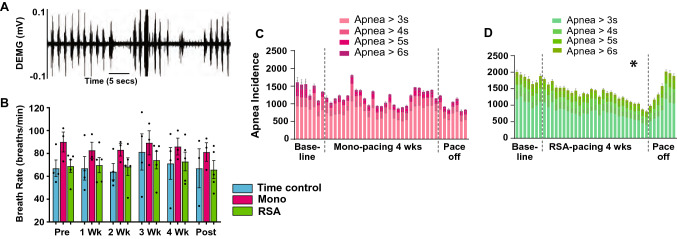
**A** A representative example of the diaphragmatic electromyographic activity (DEMG), which indicated the inspiratory phases, of a conscious sheep in heart failure. Note the instability in DEMG burst frequency including periods of apnoea. **B** Breath rate was calculated to form the burst frequency of the diaphragmatic electromyography activity (TC, *n* = 3–4, Mono *n* = 3, RSA *n* = 5). **C**, **D** The number of apneas were totaled over 24 h periods for each week of the 6 week protocol. Apnoea incidence of different length was calculated and represented as apnoeas > 3, 4, 5 and 6 s for RSA paced animals (**D**; *n* = 5) and monotonically paced sheep (**C**; Mono; *n* = 3). DEMG diaphragmatic electromyogram. 1-way ANOVA; *P < 0.05. Pace-off data not included in statistical analysis

Our heart failure sheep displayed apnoeas of 3–6 s in duration (Fig. 4A), which were quantified per 24 h (Fig. 4C, D). RSA but not Mono pacing, reduced apnoea incidence (47 + 9.8%; Fig. 4D; *P* < 0.001); this did not occur immediately but followed after the significant increase in cardiac output during the fourth week of pacing. Breath rate (breaths per min) was not different between groups, or across the different pacing protocols.

### Reduced plasma brain natriuretic peptide after respiratory modulated pacing

There was no difference in BNP concentration between RSA and Mono groups pre-pacing (6.16 ± 1.3 *versus* 6.74 ± 2.6 pmol/L, respectively) but after 4 weeks of pacing BNP was reduced in the RSA group only (RSA: 5.16 ± 0.7; Mono 6.34 ± 1.7, pmol/L; *P* = 0.066). Next, we asked whether the tissue damage caused by heart failure was reversed by RSA pacing.

### Cardiomyocyte structure after RSA pacing

**Fig. 5:**
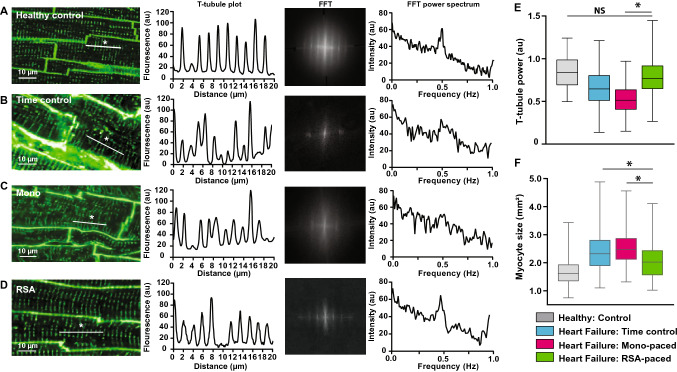
T-tubule morphology and myocyte size were measured from cells labelled with the cell membrane stain wheatgerm agglutin (WGA). **A**–**D** Representative images of the lateral wall of the left ventricle from a healthy (control), heart failure (time control), Mono and RSA paced heart failure sheep, respectively. For each sheep group, T-tubule fluorescence intensity, a Fourier Transformation (FFT), and power spectrum of the FFT were plotted from 20 µm segments of heart cells (examples indicated with an *) are shown. T-tubule structures display a 0.5 Hz frequency (FFT power spectrum), the amplitude of the 0.5 Hz peak demonstrates their presence and uniformity. **E** T-tubule uniformity is reduced in both time control and Mono paced sheep but T-tubules are re-instated after RSA pacing and not different to healthy controls. Number of animals and cells were: (healthy control, *n* = 5 sheep, 176 cells; Time control, *n* = 5 sheep, 128 cells; Mono *n* = 4 sheep, 153 cells; RSA *n* = 3 sheep, 93 cells). **F** RSA pacing reduced cardiomyocyte cell size relative to time control and Mono paced sheep. Cell size was calculated as cross-sectional area. Data analysed included multiple animals and cell numbers: healthy controls (*n* = 5 sheep, 164 cells), time controls (*n* = 5, 174 cells), Mono (*n* = 4, 229 cells), RSA (*n* = 3, 158 cells)

T-tubule structure labelled with wheatgerm agglutin, were quantified using power spectral analysis. Heart failure disrupted the T-tubules in the left ventricle (LV) making them discontinuous and reduced their occurrence (Fig. 5B) relative to a healthy heart. Impressively, after RSA pacing (Fig. 5E), the uniformity of T-tubule structure was greatly restored relative to time controls (Fig. 5D, E), and Mono paced sheep (Fig. 5D, E; *P* < 0.05) and not different to healthy controls (Fig. 5D, E). Additionally, RSA pacing reduced myocyte hypertrophy (a hallmark of heart failure [17]) compared to hearts from time control and Mono pacing groups, and was now comparable to healthy heart tissue (Fig. 5F; *P* < 0.05). There was no difference in heart or lung weights between these groups (Table 1).

### Inducing heart rate variability without coupling to breathing

**Fig. 6:**
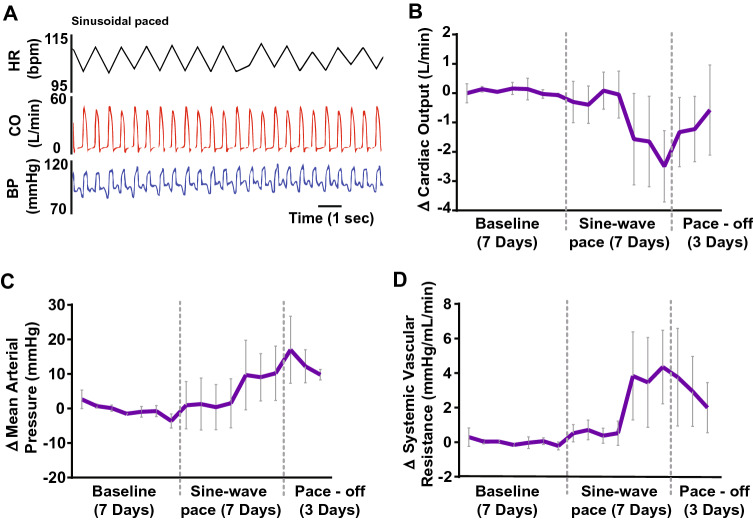
Three sheep in heart failure were paced with a sinusoidal pacing regimen that was not phase locked to respiration. **A** Representative raw data trace showing sine-wave pacing, which was generated using a grass-stimulator. Note the free-running cyclic changes in heart rate (HR); the amplitude of these oscillations was approximately 12 beats per minute and matched the RSA pacing peak-to-trough amplitude. Cardiac output (CO) and blood pressure (BP) in addition to HR are shown. **B–F** Cardiac output, mean arterial pressure and systemic vascular resistance, respectively, where data points represent 24 h average changes from pre-pacing baseline. Note the severe decline in cardiac output

To validate whether the coupling of heart rate to breathing was critical for elevating cardiac output, we used a sinusoidal pacing regimen independent of breathing (Fig. 6A). The peak to trough of the sinusoidal pacing was 12 bpm superimposed on a mean heart rate of 112 ± 5 bpm, which was identical to the settings for RSA pacing. Unlike RSA pacing, seven days of sinusoidal pacing caused a reduction in cardiac output (− 2 L/min) coupled with an increase in mean arterial pressure and systemic vascular resistance (Fig. 6B–D). At this time, one animal was euthanized as cardiac output had dropped precipitously to detrimentally low levels; in the other two sheep pacing was terminated and cardiac output returned towards baseline over the subsequent three days (Fig. 6B). Given the detrimental effects of sinusoidal pacing to the welfare of the animal, these studies were discontinued.

## Discussion

Our findings have led us to three major conclusions: (1) Reintroduction of RSA in heart failure significantly increases cardiac output, in part by reducing systemic vascular resistance. (2) RSA pacing in heart failure leads to a slow significant reduction in apnoea incidence over time. (3) Reintroduction of RSA over 4 weeks leads to a restoration of cardiomyocyte size as well as t-tubule morphology towards those of healthy controls.

### Functional role of RSA

The true role of RSA in cardiorespiratory physiology has long been debated and remains controversial. A number of benefits of RSA have been proposed, including optimizing gas exchange, stabilizing blood pressure, and more recently, mathematical modelling has suggested increasing cardiac energy efficiency [7, 39]. Although RSA has been studied extensively, its physiological importance remains equivocal [15]. In our study, we have shown that RSA pacing is distinct to conventional, metronomic or atrial sensing pacing in that it increased cardiac output by 1.25–1.95 L/min (variability in increase between animals), which corroborates a previous modelling study [7] and experimental study on rodents [39]. There was a lowering of plasma BNP levels, repair of the T-tubule structure and reduction of myocyte size that are all consistent with reverse re-modelling.

Plasma brain natriuretic peptide (BNP) is released from the ventricles in response to increased ventricular pressure/stretch, correlated with heart failure severity [47], and treatment of heart failure is clinically guided by plasma BNP [56]. Our data indicate that this marker of heart failure was reduced by re-introduction of RSA. In addition, apnoeas are associated with heart failure and contribute to low-quality life scores [33] as well as worsen survival outcome. The incidence of apnoeas was also reduced with RSA pacing. The reduction in apnoeas that occurred after improvement in cardiac output may reflect the improved blood flow to the carotid bodies and brain as shown previously [26].

Autonomic imbalance is a negative prognostic indicator in heart failure [53]. Increased sympathetic nerve activity and baroreflex resetting have been reported across clinical and animal heart failure studies [49, 58]. Our results indicate that RSA pacing reduces cardiac afterload by lowering total peripheral resistance, resets the baroreflex to operate over a lower pressure range and improves autonomic balance all of which are positively correlated with survival in heart failure [3, 12, 23]. Thus, based on the evidence herein, RSA is crucial for optimizing cardiac contractility. Given the improvement in cardiac output took several days to develop, we propose that the mechanism involves activation of intracellular signalling pathways inducing transcription to induce the reverse re-modelling of T-tubule fragmentation and cardiomyocyte hypertrophy.

Of interest, the beneficial changes observed with RSA pacing, occurred when the heart was paced with respiratory modulation for approximately 40% of the time. The lower pacing efficacy in the RSA pacing group occurred over extended periods of time when respiration rate increased to a level that the pacing device was unable to vary heart rate in phase with respiration. This is similar to humans where RSA is most prominent during non-REM sleep and during periods of rest, and lost during activity [6, 35]. This current study corroborates and significantly advances previous studies done on RSA pacing in heart failure rodents [39]. The previous study in rodents included assessment of cardiac output under anaesthesia whereas this present study directly recorded cardiac output 24 h of the day. We were also able to re-instate RSA for 4 weeks compared to 2 weeks in the rodent study and make continual real time measurements essential for revealing the time course of the response. This current study provides clinically translatable data, and a greater depth of understanding of the role RSA can have in heart failure while providing complimentary evidence in the reproducibility of these results, including the improvements in cardiac output and fractional shortening in both models.

### Variable-rate pacing versus RSA pacing

There is good evidence that a reduction in heart rate variability is associated with worse outcomes in patients with heart failure. We wanted to ascertain if the variability in heart rate needed to be entrained to the respiratory cycle or whether variable heart rate pacing alone would improve outcomes. We tested this by undertaking sinusoidal pacing of heart rate that was not linked to the respiratory cycle. Surprisingly, in all animals tested, sinusoidal pacing led to a dramatic decrease in cardiac output and pacing needed to be stopped within a week. These results indicate that the variable rate pacing needs to be entrained to the respiratory cycle for the improvement in cardiac output to occur.

### The mechanisms by which RSA can improve cardiac function

The mechanisms whereby RSA induces or inhibits transduction pathways is currently unclear. Cyclic stretch of isolated cardiomyocytes, and cardiomyocyte-derived stem cells have been shown to induce the alignment of proteins required for efficient excitation–contraction-coupling [48], induce hypertrophy [40], and upregulate mRNA expression [46]. Cyclic stretch has also been shown to activate a number of ubiquitous intracellular signalling molecules, including protein kinase C, and MAPK, which may be involved in the observed reverse remodelling [41]. However, the relationship between cyclic stretch and rhythmic changes and how this effect gene expression is currently unknown. Our data that sinusoidal pacing not coupled with respiration did not lead to the same improvement in cardiac function suggests that the stretch needs to be aligned to the respiratory cycle and the sequalae of effect on intra-pulmonary and pleural pressures affecting venous return to left and right atria. It is known that RSA arises via alterations in cardiac vagal tone. Our study suggests even in the absence of changes in autonomic tone, the mechanical effects of RSA induced by direct electrical pacing of the left atria is important in modifying heart function. This current study protocol cannot determine if the reverse remodelling precedes the improvement in cardiac function or vice-versa. One way to establish this would be sequential biopsies from the RSA paced sheep at specific time points during the increase in cardiac output.

We have previously shown that this model of heart failure has a higher resting heart rate than equivalent healthy controls [4]. This is consistent with the well-established activation of the renin-angiotensin system and the sympathetic nervous system in heart failure [5, 59]. Importantly all three HF groups did not have any significant differences in their baseline heart rates before that start of pacing or the time control period. We must note that the RSA paced group were paced to a comparable mean rate as the monotonic group but in this scenario resulted in improved cardiac function. There were no significant differences in arterial pressure during the pacing in either group although systemic vascular resistance increased in the monotonic group. It is possible that this increase in vascular resistance may have contributed to the decline in cardiac function in the monotonic pacing group.

### Is there a clinical need for a novel RSA pacemaker?

Heart failure affects more than 26 million people globally, and is a growing pandemic [10]. Around 50% of those with heart failure will die within 5 years [32]. There remains no cure although medication and devices delay progression. Traditional pharmacotherapy for heart failure with reduced ejection fraction includes blockers of angiotensin-converting enzyme, beta-adrenoceptors and mineralocorticoid receptors which typically improve ejection fraction by 5–7% and life longevity [21, 44]. More recent drugs such as sacubitril/valsartan (Entresto) and dapagliflozin have utility in refractory patients [30, 31] but the improvements in ejection fraction remain modest [25]. In addition to pharmacotherapy, pacemakers are fitted to a heterogeneous population of patients with heart failure experiencing chronotropic incompetency, conduction failure, arrhythmias, and dyssynchronous ventricles. Standard pacemakers do not provide clinical benefit in all patients and can worsen heart failure in some patients [42]. One option is cardiac resynchronization therapy (CRT). While the absolute mean increase in ejection fraction achieved by biventricular pacemakers which incorporate CRT is within the 5–11% range [11, 29, 34, 45], there is wide variability in the extent of left ventricular remodelling and improvement in LVEF. The best responding patients termed super-responders show an increase in LVEF of > 14.5% (mean LVEF increase 17.5 ± 2.7%) [24] with some displaying near-normalisation of EF [16] indicating patient selection may be crucial for optimal use of CRT. It must be noted that both left bundle branch block and having no prior myocardial infarction are predictors of these super-responders which means that CRT does not meet the needs of all patients. The benefits tend to be limited to patients with intraventricular conduction delay (prolonged QRS duration), which only applies to around 20% of heart failure patients [51] and heart failure can worsen in patients who have a normal QRS duration [50]. Moreover, 30% of patients fitted with CRT devices fail to respond [13].

The 20% improvement in cardiac output seen with RSA pacing represents an impressive improvement in cardiac function. It is important to note that RSA is minimal or abolished in many patients with heart failure [17]. So even when atrial sensing is used in current pacemakers, RSA cannot be reinstated. In addition, while many current pacemakers are rate-adaptive where pacing frequency increases in response to physical activity or an elevated respiratory frequency (calculated over an epoch of time), in all cases they cannot re-instate RSA. In summary, current treatments (pharmacological or device based) when effective are limited and current therapy is ineffective in a substantial number of heart failure patients. Thus, additional therapeutic options are urgently needed, and we propose that this clinical gap might be addressed with RSA pacing.

Given the dramatic improvements in cardiac output, we wanted to determine the effects on systemic vascular resistance. The length of this protocol meant that conscious chronic nerve recordings were not possible in this study. However, we have calculated systemic vascular resistance from direct recordings of cardiac output and mean arterial pressure and this indicates reintroduction of RSA pacing is associated with a significant reduction in systemic vascular resistance. Together with this, our direct recordings of renal sympathetic nerve activity indicate that baroreflex control of sympathetic nerve activity to the kidney is improved. Although this study was performed in a clinically relevant translational model, it must be noted that the animals in this study were not on common heart failure medication. Given that RSA is mediated by the parasympathetic nervous system which is not targeted by current medications, we anticipate that re-introducing RSA would still be beneficial in a patient population with contemporary medication on board.

## Conclusions

Our work identifies for the first time, in a large animal translational animal model that reintroducing respiratory modulated pacing in heart failure results in a dramatic improvement in directly recorded cardiac output. In addition, RSA pacing reduces apnea incidence and re-models cardiomyocyte morphology. Taken together, our data support the concept of reintroducing RSA as a pacing paradigm in pacemakers.
